# Unicameral (simple) and aneurysmal bone cysts: the effect of insufficient curettage on recurrence

**DOI:** 10.11604/pamj.2016.24.311.9624

**Published:** 2016-08-16

**Authors:** Suat Çelik, Abuzer Uludağ, Hacı Bayram Tosun, Sancar Serbest, Murat Gürger, Sabahattin Kılıç

**Affiliations:** 1 Department of Orthopaedics and Traumatology, Faculty of Medicine, Adiyaman University, Adiyaman, Turkey; 2 Department of Orthopaedics and Traumatology, Faculty of Medicine, Kırıkkale University, Kırıkkale, Turkey; 3 Department of Orthopaedics and Traumatology, Faculty of Medicine, Fırat University, Elazığ, Turkey

**Keywords:** Unicameral bone cysts, aneurysmal bone cysts, curettage, healing

## Abstract

**Introduction:**

Curettage of the cyst and bone grafting are the most common methods used in the treatment of unicameral bone cysts (UBC) and aneurysmal bone cysts (ABC). Recurrence of these cysts is often associated with insufficient curettage of the cyst during surgery. In this study, we aimed to evaluate the effect of insufficient curettage on recurrence in patients with UBC and ABC.

**Methods:**

The retrospective study included 18 patients with UBC and 14 patients with ABC that were surgically treated by curettage and bone grafting in our clinic between 2006-2013. Mean age was 19.80 (range, 4-50) years in the patients with UBC and 21.76 (range, 4-56) in the patients with ABC. The diagnosis of the cysts was established both clinically and radiologically. Mean follow-up period was 36 (range, 6-60) months both in the patients with UBC and ABC. The patients with recurrence underwent a second curettage and grafting procedure. Healing and recurrence were evaluated according to modified Neer's scale.

**Results:**

Recurrence occurred in 8 patients. Of these, 5 patients underwent a second curettage and grafting procedure and 3 patients were lost to follow-up. Complete healing occurred in all the patients that underwent a second curettage and grafting procedure.

**Conclusion:**

The achievement of complete healing in the patients that underwent a second curettage and grafting procedure indicates that the recurrence of UBC and ABC is associated with insufficient curettage.

## Introduction

Unicameral and aneurysmal bone cysts are benign bone tumors commonly seen in the first two decades of life, particularly between the ages of 4 and 10 [1, 2]. Unicameral bone cysts (UBC), also known as simple bone cysts, mostly arise in the humerus (50%), followed by the femur and the tibia [3–5]. UBC are usually located in the metaphyses of long bones and extend into the physis, and may rarely cross the physis and extend into the epiphysis [6, 7]. Radiologically, UBC are radiolucent lesions surrounded by a thin cortical border, and they often expand symmetrically. Although their exact prevalence remains unknown, UBC account for 3% of all bone lesions [8, 9]. Aneurysmal bone cysts are benign, expansile lytic lesions of the bone that are also located in the metaphyses of long bones. Aneurysmal bone cysts (ABC) account for 1-6% of all primary bone tumors detected in biopsy [10–13]. The treatment of UBC and ABC includes the curettage of the fibrous membrane in the cyst wall and bone grafting, injection of steroid, grafting with autologous bone marrow, drainage of the cyst with insertion of intramedullary implant or kirschner wires without opening of the cortical window, and total or subtotal resection of the cyst. In this study, we aimed to evaluate the effect of insufficient curettage on recurrence in patients with UBC and ABC.

## Methods

**Figure 1:**
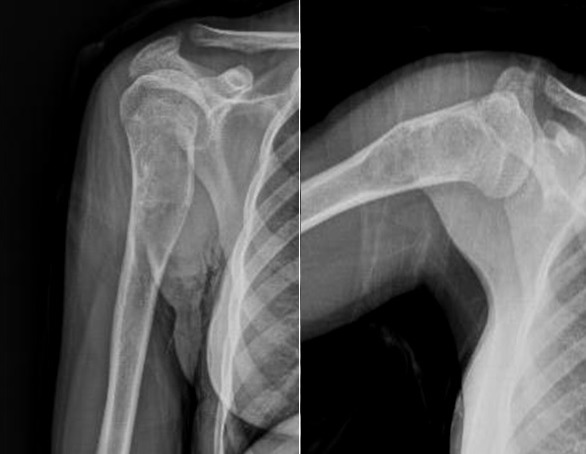
Radiography view of the simple bone cyst of the proximal humeral in 13 year old boy

**Figure 2:**
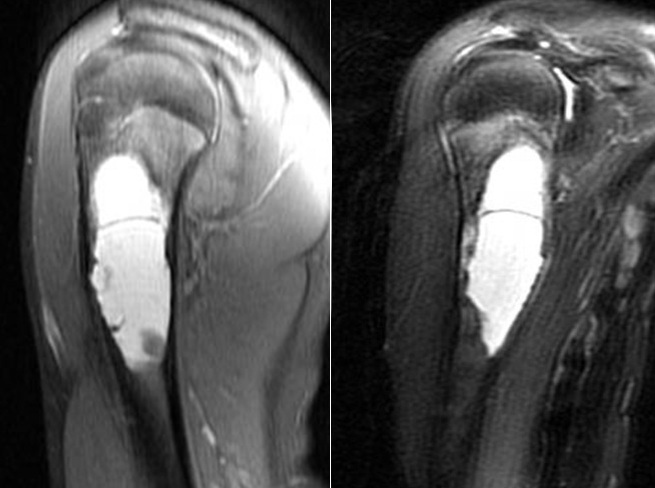
T2-weighted sagittal MRI depicting of the lesion

**Figure 3:**
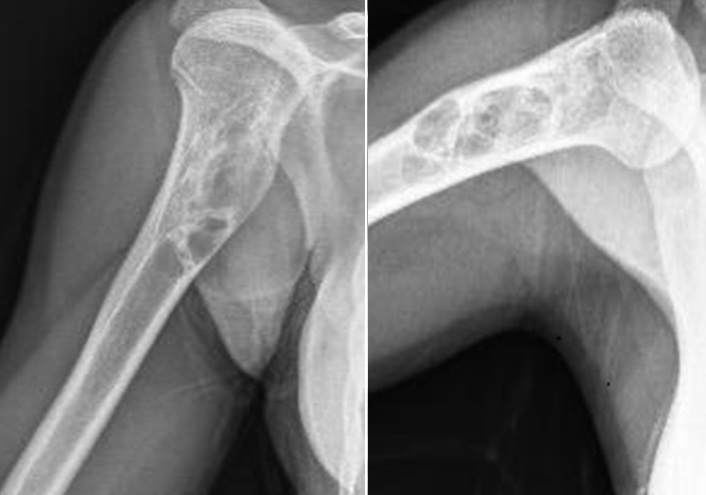
Radiography view of the tumour recurrence in one year after surgery

**Figure 4:**
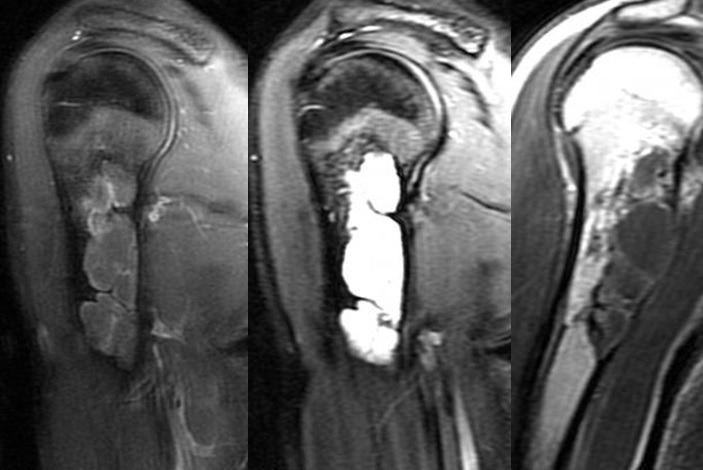
MRI imaging of the recurrence

**Figure 5:**
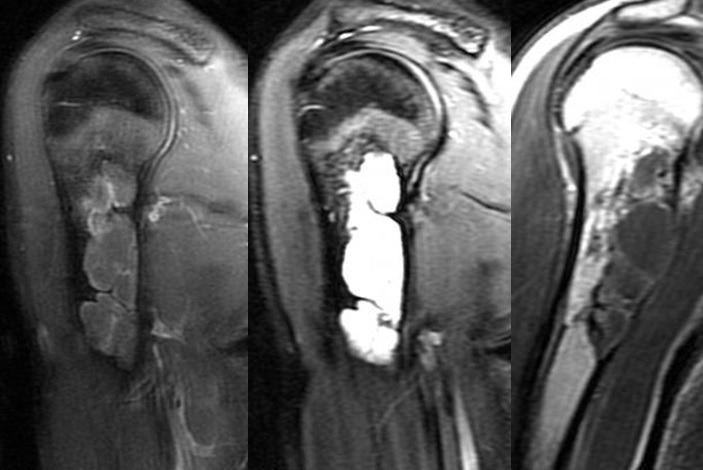
Complete healing after a second curettage and grafting procedure

The retrospective study included 18 patients with UBC and 14 patients ABC who were surgically treated by curettage and bone grafting in our clinic between 2006-2013. Three patients with recurrent bone cysts including 2 UBC and 1 ABC were excluded from the study. Therefore, 16 patients with UBC and 13 patients with ABC were included in the study. Moreover, the cysts treated with other treatment methods (decompression alone, corticosteroid injection, cryotherapy with liquid nitrogen or phenol, and bone cement after curettage) and the patients with pathological fractures were also excluded from the study. An informed written consent was obtained from each patient. An approval was obtained from the Local Ethics Committee for Biomedical Research (29.03.2012-07/01). All the patients were evaluated by direct radiography and magnetic resonance imaging (MRI) prior to surgery. The diagnosis of the cysts was established both clinically and radiologically. All the patients underwent curettage of the cyst and bone grafting under general anesthesia. Curettage was performed by creating a cortical window in the center of the cyst, sufficient enough to view the entire cavity. Following the curettage of the membrane in the cyst, allografting was performed in the cyst cavity. Internal fixation was performed in the patients with a risk of fracture, and cast-splint was performed in patients with no risk of fracture. The patients were followed up at months 1, 2, 3, 6, and annually. At the follow-up period, healing and recurrence were evaluated depending on the rate of the filling of the cystic area with bone formation (Figure 1, Figure 2, Figure 3, Figure 4, Figure 5). The rate of the filling of the cystic area was graded according to the modified Neer's scale by Chang et al. [14] (Table 1).

**Table 1:** Modified Neer’s scale

Complete Healing	Cyst filled [more than 95%) by formation of new bone with or without small static, radiolucent area[s) less than 1 cm in size
Healing with defect	Static, radiolucent area[s) less than 50% of the diameter of the bone with enough cortical thickness to prevent fracture
Persistent cyst	Radiolucent areas greater than 50% of diameter of the bone and with a thin cortical rim. No increase in cyst size
Recurrent cyst	Cyst reappeared in a previously obliterated area or a residual radiolucent area has increased in size

## Results

Thirteen (65%) patients with UBC and 12 (60%) with ABC were aged below 20 years. Mean age was 19.80 (range, 4-50) years in the patients with UBC and 21.76 (range, 4-56) in the patients with ABC. Mean follow-up period was 36 (range, 6-60) both in the patients with UBC and ABC. No significant difference was observed between the two groups in terms of age and follow-up period. Recurrence occurred in 5 out of 18 patients with UBC. Of these, 3 patients underwent a second curettage and grafting procedure. The other 2 patients were excluded from the study because they were lost to follow-up. Internal fixation was performed in 7 out of 18 patients with UBC due to the risk of fracture. In one patient with UBC that did not undergo internal fixation, a second curettage and grafting procedure was performed due to recurrence. Internal fixation was performed in 2 out of 5 patients with recurrence. One of these patients, who underwent plate-screw osteosynthesis and developed recurrence, underwent a Ender type of intramedullary nailing following a second curettage and grafting procedure, after the removal of the implant. In the other patient, who also developed recurrence, the intramedullary kirschner wire was removed, and plate-screw osteosynthesis was performed following a second curettage and grafting procedure. Complete healing was observed in all the patients that underwent a second curettage and grafting procedure due to recurrence. No additional internal fixation was performed in 14 patients with ABC. Of these, recurrence occurred in 3 patients. In 2 of these patients, a second curettage and grafting procedure was performed and complete healing was achieved. The other patient was excluded from the study because the patient was lost to follow-up.

## Discussion

There are several treatment methods for unicameral bone cysts (UBC) and aneurysmal bone cysts (ABC). However, these methods have been shown to cause recurrence at varying rates, except for total resection of the cyst [**3**]. Curettage and grafting are one of the most common techniques used in the treatment of UBC and ABC [2, 15–17]. Ruiter et al. [16] and Mankin et al. [17] reported recurrence in 34.2% and 20% of the patients that underwent curettage, respectively. Campanacci et al. [18] treated 91 of the 198 patients with ABC and reported recurrence in 19 (20.8%) of the patients. In our study, recurrence occurred in 3 (18.75%) out of 14 patients with 14 ABC. Curettage plus bone grafting is a commonly performed surgical procedure in the treatment of UBC. This procedure has been shown to have a recurrence rate of 22-47% [19, 20]. Sung et al. [21] performed curettage plus grafting by creating a cortical window in 39 out of 167 patients with UBC and reported that 25 (64%) of the patients experienced failed treatment. They claimed that the differences in failure rates in their study versus published rates might be attributed to the longer follow-up periods in their study, which might have resulted in detection of more late failures. Kokavec et al. [22] also performed curettage plus bone grafting in 19 patients with UBC and reported that complete healing was achieved in 16 and recurrence occurred in 3 patients. Hagmann et al. [23] reported that recurrence occurred in 37% of the patients that underwent curettage plus bone grafting and no significant difference was observed between the groups that underwent autografting and allografting in terms of recurrence. Corticosteroid injection, curettage plus bone grafting, and decompression of the cyst are commonly used in the treatment of ABC [19, 20]. Glowacki et al. [24] compared 132 patients with ABC that underwent curettage plus bone grafting or steroid injection. They reported that the healing rate was higher in the patients that underwent curettage plus bone grafting compared to the patients that underwent steroid injection, but no significant difference was observed between cyst size and recurrence. Brecelj et al. [25] reported that successful treatment was achieved in 50% of the patients that underwent curettage plus bone grafting, in 19% of the patients treated with corticosteroid injection, and in 65% of the patients treated with decompression. Alemdar et al. [26] compared the patients that underwent conventional surgical treatment or corticosteroid injection and found that conventional surgical treatment yielded better results compared to corticosteroid injection in terms of duration and quality of healing and the healing rate. The study also suggested that decompression is one of the key components of surgical treatment. In our study, curettage plus bone grafting with allograft was performed in all of the 18 patients, and recurrence occurred in 5 (27.7%) patients. Recurrence is associated with the presence of residual tumor tissue caused by insufficient curettage [15, 17]. Gibbs et al. [27] reported that the recurrence rate was decreased to 12% by the use of contemporary methods of curettage with a high-speed burr and suggested that this method should be the method of choice for preventing residual tumor tissue after curettage. The size of the cortical window created for curettage is of prime importance for the prevention of residual tumor tissue after curettage. Cole et al. [28] performed curettage in 25 child patients and reported that multiple tumor tissues were remained in the lesions due to the small size of the cortical window. The study suggested that the size of the cortical window is an important factor for a successful curettage procedure. Dormans et al. [29] also suggested that the size of the cortical window has a direct effect on the success of the curettage and the visibility of the cystic cavity. Adjuvants such as phenol [30], liquid nitrogen [31], and bone cement [32] are also used for the prevention of recurrence after curettage. Of these, bone cement is the most commonly performed method in the prevention of recurrence. This method exerts an adjuvant effect by creating a certain degree of temperature, which should be 50° C or higher. Bone cement is advantageous over the other adjuvants since it is not only practical but also supports the cystic area. Ozaki et al. [32] performed bone cement in 35 and grafting in 30 patients after curettage and reported that recurrence occurred in 17% of the patients administered with bone cement as opposed to 37% of the patients administered with grafting. Recurrence is more common in children than in adults. A previous study investigated 7 children with a mean age of 5.5 years and reported that recurrence occurred in 5 patients within the 8 months after surgery and the patients were reoperated [33]. Ramirez et al. [34] treated 53 patients with ABC and reported that recurrence was mostly seen in children aged below 12 years. The high rates of recurrence in children versus adults has been shown to be associated with the aggressive progress of the disease in these age groups and the presence of residual tumor tissue associated with insufficient curettage caused by the proximity of the cyst to the open physeal line. In our study, 3 out of 5 of the patients with recurrence were aged between 4-10 years. We consider that the high rate of recurrence seen in children is associated with the avoidance of aggressive curettage. Surgical resection of the cyst has the lowest rate of recurrence in the treatment of ABC. Campanacci et al. [18] and Arlet et al. [35] performed surgical resection and reported that no recurrence occurred in any patient. Since resection is an aggressive surgical procedure, the use of resection should be avoided in the regions where resection may lead to functional complications [36]. The periods with the highest frequency of recurrence in benign bone cysts and the durations of follow-up required for the recurrence of these cysts are important issues for the treatment of these cysts. A previous study investigated 66 patients with ABC and reported that recurrence occurred in 26 patients within the first 4 years. The study also reported that recurrence occurred within the first year in 2>4 out of 26 patients [2]. Similarly, Clough et al. [36] and Freiberg et al. [33] reported that recurrence occurred within the first 1 year and the first 8 months, respectively. In contrast, Dios et al. [2] reported that recurrence occurred in 19% of the 153 patients treated with curettage within the first 2 years. In patients with UBC, similar to patients with ABC, recurrence often occurs within the first two years and requires long follow-up periods. Cho et al. [37] reported that recurrence occurred within the first 13 months, whereas Teoh et al. [38] reported an average time of recurrence of 10 (range, 4-27) months. In our study, recurrence occurred within the first year in 3 out of 5 patients. Recurrence occurred within the six months in 2, at the first year in 1, at the 4th year in 1, and at the 5th year in 1 patient. We believe that although recurrence is most likely to occur within the first 2 years, recurrence should be kept in mind in the follow-up periods after the first 2 years as well. Therefore, we suggest that the patients undergoing surgical treatment should be followed up and informed about their progress each year.

## Conclusion

The treatment of aneurysmal and unicameral cysts remains controversial. However, complete curettage of the cyst and bone grafting are useful methods to be used in the treatment of these cysts. Insufficient curettage may lead to residual tumor tissues, resulting in recurrence. Adjuvant therapies may be added to surgical treatment when needed. The patients undergoing surgery due to aneurysmal or unicameral cysts should be closely monitored within the first two years after surgery since recurrence is most likely to occur within this period.

### What is known about this topic

- Recurrence of unicameral and aneurysmal bone cysts is often associated with insufficient curettage of the cyst during surgery;
- Recurrence of these cysts often occurs within the first two years and requires long follow-up periods;
- The high rates of recurrence in children versus adults has been shown to be associated with the aggressive progress of the disease in these age groups and the presence of residual tumor tissue associated with insufficient curettage caused by the proximity of the cyst to the open physeal line.

### What this study adds

- Recurrence of unicameral and aneurysmal bone cysts is often associated with insufficient curettage of the cyst during surgery;
- Recurrence of these cysts often occurs within the first two years and requires long follow-up periods;
- The high rates of recurrence in children versus adults has been shown to be associated with the aggressive progress of the disease in these age groups and the presence of residual tumor tissue associated with insufficient curettage caused by the proximity of the cyst to the open physeal line.
